# TEMPRO: nanobody melting temperature estimation model using protein embeddings

**DOI:** 10.1038/s41598-024-70101-6

**Published:** 2024-08-17

**Authors:** Jerome Anthony E. Alvarez, Scott N. Dean

**Affiliations:** Naval Research Laboratory, Center for Bio/Molecular Science and Engineering, Washington, DC USA

**Keywords:** Antibodies, Nanobodies, Single-domain antibodies, Proteins, Machine learning, Neural networks, Thermostability, Computational biology and bioinformatics, Computational models, Machine learning, Protein analysis

## Abstract

Single-domain antibodies (sdAbs) or nanobodies have received widespread attention due to their small size (~ 15 kDa) and diverse applications in bio-derived therapeutics. As many modern biotechnology breakthroughs are applied to antibody engineering and design, nanobody thermostability or melting temperature (T_m_) is crucial for their successful utilization. In this study, we present TEMPRO which is a predictive modeling approach for estimating the T_m_ of nanobodies using computational methods. Our methodology integrates various nanobody biophysical features to include Evolutionary Scale Modeling (ESM) embeddings, NetSurfP3 structural predictions, pLDDT scores per sdAb region from AlphaFold2, and each sequence’s physicochemical characteristics. This approach is validated with our combined dataset containing 567 unique sequences with corresponding experimental T_m_ values from a manually curated internal data and a recently published nanobody database, NbThermo. Our results indicate the efficacy of protein embeddings in reliably predicting the T_m_ of sdAbs with mean absolute error (MAE) of 4.03 °C and root mean squared error (RMSE) of 5.66 °C, thus offering a valuable tool for the optimization of nanobodies for various biomedical and therapeutic applications. Moreover, we have validated the models’ performance using experimentally determined T_m_s from nanobodies not found in NbThermo. This predictive model not only enhances nanobody thermostability prediction, but also provides a useful perspective of using embeddings as a tool for facilitating a broader applicability of downstream protein analyses.

## Introduction

Nanobodies are small antibody fragments derived from the unique small heavy-chain antibodies found in camelids (such as camels, llamas, and alpacas), consisting of only heavy chains, unlike conventional antibodies. Nanobodies are the variable domains of these heavy-chain antibodies and are known as VHH fragments. These single-domain antibodies retain the ability to specifically bind to antigens, similar to conventional antibodies, but they are much smaller (about 12–15 kDa compared to 150 kDa for full antibodies). The applications potential of nanobodies are varied. As therapeutics, these antibodies can be used to target and neutralize pathogens or to block disease-related proteins. Their small size allows for better tissue penetration, making them effective for treating diseases such as cancer, inflammatory conditions, and infectious diseases^1,2^. As diagnostics, nanobodies can be employed in diagnostic assays due to their high specificity and affinity for target molecules. They can be used in imaging techniques to detect disease markers or in biosensors to provide rapid and accurate diagnostics. Further, they can be used in basic research to investigate protein–protein interactions, localize proteins within cells, and isolate proteins of interest via affinity purification. And, in detection, nanobodies can be applied to control plant diseases or as biosensors to detect pathogens and contaminants in a variety of products^3,4^. With this wide array of applications, the interest in antibody design and engineering remains strong and its growth is being propelled further by the increase in interest in generating synthetic proteins with desired properties through deep learning, such as generative artificial intelligence, which has demonstrated some success^5^.

### Nanobody characteristics

**Figure 1:**
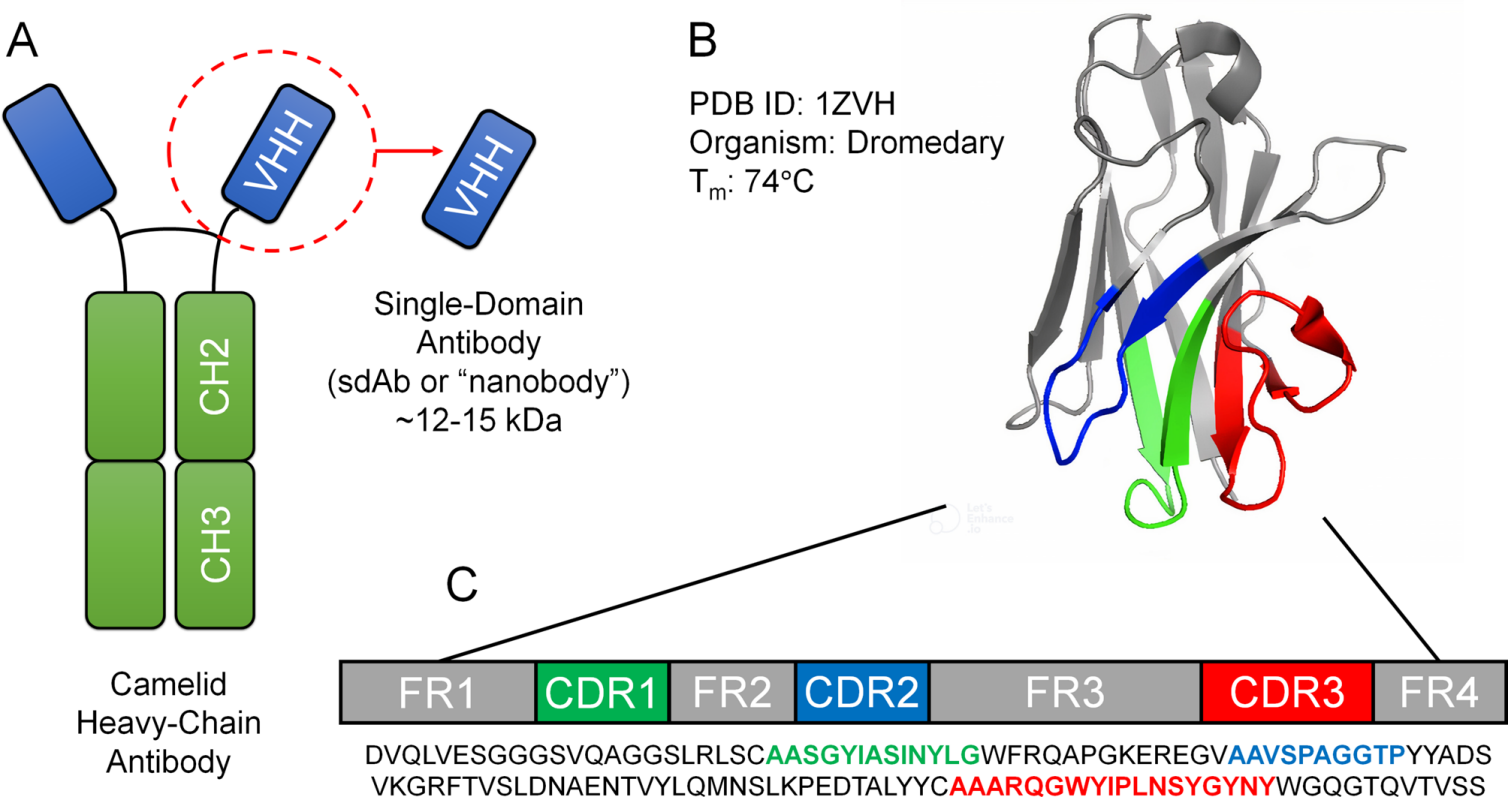
Nanobody structure and sequence characteristics. (**A**) Representation of a camelid heavy-chain antibody with VHH encircled in red. (**B**) Representative nanobody from PDB (ID: 1ZVH)—crystal structure of VHH domain from organism *camelus dromedaries*—CDR regions are highlighted in green (CDR1), blue (CDR2), and red (CDR3). (**C**) Sequence arrangement of a nanobody. CDR sequences are highlighted with the same color scheme.

One of the first studies in nanobodies was conducted in 1989 when the isolation of stable mouse antibody VH domains that could bind antigens with relatively high affinity reported by Ward et al.^6^. In emergence from these studies, variable heavy-chain antibodies (VHHs) were found in camels in 1993 by Hamers-Casterman et al.^7^ which represent the smallest naturally derived antigen-binding fragments. The structure of immunoglobulins consists of four chains: two identical light chains and two identical heavy chains which corresponds to the well-known Y shape of an antibody (Fig. 1A). Single variable domain on a heavy chain (sdAb) structural characteristic pertains to the fragment of an antibody consisting of a single monomeric variable domain separate from its light chain. VHHs contain 4 framework regions (FRs) that form the core structure of the immunoglobulin domain and 3 complementarity-determining regions (CDRs) that are involved in antigen binding (Fig. 1B,C). FRs are conserved regions of the antibody which allow the antigen-binding, hypervariable CDR regions to be stable^8^; these are summarized in Table 1.

**Table 1:** Roles of framework (FR) and complementarity-determining regions (CDR).

Region	Description
FR1	Framework Region 1 (N-terminus) Possible target of efficient mutagenesis for generating a variety of affinity-matured scFv mutants^9^
CDR1	Complementarity-determining Region 1 4 residues after first cysteine 6–15 residues Extended hypervariable CDR1 region (residues 27–30) is used together with CDR3 to increase surface area interacting with the antigen, and thus proposed to vary these amino acids for synthesis to increase potential antigen binding^10^
FR2	Framework Region 2 Dromedary VHHs have an extended CDR3 that is often stabilized by an additional disulfide bond with a cysteine in CDR1 or FR2^11^
CDR2	Complementarity-determining Region 2 10 to 20 residues after end of CDR1 8–15 residues Can provide additional antigen interaction force with CDR3^12^ and an unusual extra glycine residue recently demonstrated a neutralizing spot for toxin binding^13^
FR3	Framework Region 3 Introducing a non-canonical disulfide bond into the hydrophobic core of llama VHHs between FR2 and FR3 proved to increase thermal stability at neutral pH and resistance to proteolytic degradation^14^
CDR3	Complementarity-determining Region 3 30 to 50 residues after end of CDR2 3–25 residues The most variable region and considered as the center of antigen recognition^15^
FR4	Framework Region 4 (C-terminus)

### Nanobody thermostability

Nanobody thermostability or melting temperature (T_m_) is crucial for their successful utilization, in their varied applications. In their storage and transport, thermostable antibodies can be handled without refrigeration, crucial in remote or low resource areas. In the human body, thermostable nanobodies have longer half-lives and greater efficacy due to lower degradation. In biocatalysis or bioremediation, thermostable nanobodies can remain effective at higher temperatures, if needed for the application^16,17^. Because of these benefits, several approaches in estimating T_m_s of proteins have been investigated such as through molecular dynamics simulations^18^, deep learning^19,20^, multiple machine learning ensembles^21^, and even early works of using correlative composition of dipeptides^22^. However, some of these studies are generalized too broadly to proteins overall, while others only classify the predicted T_m_s on a discrete scale (e.g., > 65 °C, between 55 and 65 °C, and < 55 °C) or being thermophilic versus non-thermophilic, which contributes to their unreliability of precise T_m_ prediction specific to nanobodies.

Recently, predicted embeddings through the use of pre-trained models have been used as features for exploring biophysical properties of proteins^23^ and even DNA sequences^24^. State-of-the-art models continue to demonstrate protein representations to aid in prediction, classification, and recognition tasks. Here we introduce TEMPRO: nanobody melting Temperature Estimation Model using PROtein embeddings. This tool aims to predict the melting temperature of a protein from its sequence alone and is specifically tailored for nanobodies. Along with the evaluation of the feasibility of using protein embeddings as a predictive feature, we have benchmarked the best-performing model against other quantifiable biophysical properties of nanobodies, several other machine learning techniques, and some of the latest prediction models from literature.

## Materials and methods

### Nanobody datasets

A recently published dataset from the NbThermo database^25^ includes 548 nanobody sequences with their corresponding organism source, experimentally measured melting temperatures (T_m_s in °C), target antigen, framework (FR) and complementarity-determining regions (CDR), and digital object identifier source information. We combined this with our in-house, manually curated nanobody dataset consisting of 166 sequences from multiple published studies (all raw and processed data can be found in our GitHub page in the Data Availability Statement). The 548 nanobodies from NbThermo contain 9 sequences with missing T_m_s, thus removed in the data cleaning. Subsequently, we combined the cleaned NbThermo nanobodies and our internal nanobody dataset, and then filtered duplicate sequences into a single observation. The processed data results into 567 unique sequences with their corresponding T_m_s which was then split into 80:20 ratio for training and validation sets. All sequences were restricted to the 20 natural amino acids and sequences with non-conventional residues were excluded.

### Thermostability predictors

#### Physicochemical characteristics

Global Sequence Signature analyses (GSS) by Kunz et al.^26^ demonstrated that the residue compositions reflected in physicochemical properties including aliphatic index, charge and others contribute to nanobody stability, and thus we have included several of the same features in the model. Most of the biophysical properties quantified for the generated antibodies were generated from the Peptides R Package^27^ which computes a set of physicochemical characteristics from the amino acid sequence. Other properties were computed through in-house calculations. These characteristics are summarized in Table 2.

**Table 2:** Nanobody physicochemical characteristics and descriptions.

Metadata	Description
Length	Number of amino acids in a sequence. The average sequence length of all 567 nanobodies is ~ 124 amino acids
Aliphatic index	Index represented by the relative volume occupied by aliphatic side chain of alanine, valine, isoleucine, and leucine; regarded as a positive factor for the increase of thermostability of globular proteins^28^
Instability index	The predicted in vivo stability of a protein from its primary sequence^29^. An instability index of < 40 is considered as stable while > 40 as unstable
Hydrophobicity (GRAVY) index	GRand AVerage of hydropathY (GRAVY) index based on the KyteDoolittle scale^30^ where positive values equate to a more hydrophobic characteristic and negative for hydrophilic. The hydrophilic region in a VHH accounts for the superior stability and solubility^31^
Flexibility index	Described as the symmetric/asymmetric distribution of amino acid residues in the protein^32^. Protein flexibility link structure and function in the context of adaptation to temperature, qualitatively defined as protein’s capability to alter its conformation in response to a change in temperature^33^. Flexibility indices also correlate with the tendency of the side chain to be buried or exposed^34^
Polarizability	One of the 26 physicochemical descriptor variables discussed by Sandberg et al.^35^ that describes an amino acid’s steric properties. The protein’s spatial arrangement dictates its propensity to become polarized temporarily where higher polarizability favors more interactions. It was previously demonstrated that several cell types can contribute to antibody polarizability which results into differences in immune response and binding^36–38^
Charge	Net charge of a protein sequence based on the Henderson-Hasselbalch equation^39^ using Lehninger scale^40^. The theoretical net charge of the complementarity-determining regions (CDRs) is a strong predictor of antibody specificity or the degree to which an immune response discriminates between antigenic variants^41,42^
Tryptophan count	Mainly used as factors for computing the extinction coefficient. However, these are included as features due to intrinsic fluorescence of proteins^43^ during elevated temperature conditions (such as measuring T_m_ using circular dichroism) which have distinct absorption wavelengths^44^
Tyrosine count	
Cysteine count	Cysteine residues serve essential roles in protein structure and function by conferring stability through disulfide bond formation which maintains proper maturation and localization through protein–protein intermolecular interactions^45^. This is also important in the antibody cysteine-based conjugation in determining framework regions through numbering
Extinction coefficient	A protein’s extinction coefficient indicates how much light a protein absorbs at a certain wavelength and it is involved in protein purification^46^
Absorbance	Commonly, the optical absorption of proteins is measured at 280 nm^47^. According to the Beer–Lambert law, the concentration of a protein is directly proportional to its absorbance, at a defined wavelength and at a constant path length. This measure is also involved in structural characterization of proteins and some antibodies^48^
Molecular weight	Single-domain antibodies are composed of a variable domain of heavy chain fragments that are around 11–15 kDa^49^

#### Multi-agent stability prediction upon point mutations (MAESTRO)

MAESTRO is a structural method for predicting changes in protein stability which implements a multi-agent machine learning (ML) system, provides predicted free energy change (ΔΔG) values, and a corresponding prediction confidence estimation^50^. This confidence estimation for all 567 nanobodies were extracted and used as a predictive feature.

#### Protein surface/solvent accessibilities and structural features (NetSurfP3)

NetSurfP3 predicts solvent accessibility, secondary structure, structural disorder and backbone dihedral angles for each residue of an amino acid sequence^51^. Predictions from the NetSurfP3 tool were obtained from: https://services.healthtech.dtu.dk/services/NetSurfP-3.0/.

#### AlphaFold2 pLDDT scores with antibody numbering

Multiple protein modeling algorithms exist in literature, but tailoring a specific prediction tool for nanobodies do not always yield better model predictions. A multitude of variability also exists in the results comparing different prediction tools such as NanoNet^52^, IgFold^53^, ESMFold^54^, OmegaFold^55^, trRosetta^56^. However, it was previously shown that general protein modeling programs, such as AlphaFold2^57^, have been exposed to a wide and diverse set of protein structures which yields better predictions, especially with CDRs^58^. Therefore, with its ease of use and a well-established algorithm, all structural predictions of 567 nanobodies used in this study were predicted using AlphaFold2. In their structural predictions, they have observed high side-chain accuracy when the protein backbone prediction is accurate, and this is denoted by their predicted local-distance difference test (pLDDT) confidence measure. These confidence scores were extracted from each protein data bank (PDB) file outputs.

On the other hand, the antibody numbering used from the NbThermo database is the Aho numbering scheme^59^. We used the same numbering scheme for the other nanobodies with missing antibody annotations for consistency using ANARCI^60^. Ultimately, we have extracted the pLDDT scores for each nanobody region sequences (i.e., FRs 1–4 and CDRs 1–3).

#### Protein structure embeddings

Evolutionary scale modeling (ESM) pre-trained weights from MetaAI for transformer protein language models were utilized to predict the nanobody structural embeddings. The pre-trained weights used from their repository (https://github.com/facebookresearch/esm) are summarized in Table 3.

**Table 3:** ESM pre-trained weights.

Model	Weights	Dataset	Description
ESM-2	esm2_t33_**650M**_UR50D()	Uniref50 (UR50)^61^	General-purpose protein language model that can be used to predict structure, function, and other protein properties from sequences^54^
	esm2_t36_**3B**_UR50D()		
	esm2_t48_**15B**_UR50D()		

### Predictive models

#### Machine learning (ML) ensembles

This study used multiple machine learning ensembles and tools from the Scikit-learn Python module^62^, namely XGBoost (XGB)^63^, Random Forests (RF)^64^, Support Vector Machines (SVM)^65^, and Least Absolute Shrinkage and Selection Operator (LASSO)^66^. Each ML model training has been validated using repeated k-fold cross validation with five splits and three repetitions.

#### Deep neural networks (DNNs)

The early concept of backpropagation in artificial neural networks (ANNs) was introduced in the early work of David Rummelhart in 1986 to improve the memory of a network^67^ and now clearly represented as a class of learning techniques by LeCun et al.^68^. Specifically, this study used deep neural networks^69^ with one input, three hidden, and one output layers using the Keras Sequential API^70^ in training the nanobody thermostability predictors.

### Software

The overall model training and analyses were performed using Jupyter Notebooks (Python 3.9.15) under Anaconda Distribution. Data preprocessing was conducted using R/RStudio. All figures were plotted using Python’s Seaborn^71^ module and three-dimensional protein structural representations rendered through PyMOL software.

### Correlation calculations

Pairwise Pearson correlations were calculated between all structural and sequence properties of nanobodies.

## Results

### Exploratory analyses uncover relationships between nanobody properties

**Figure 2:**
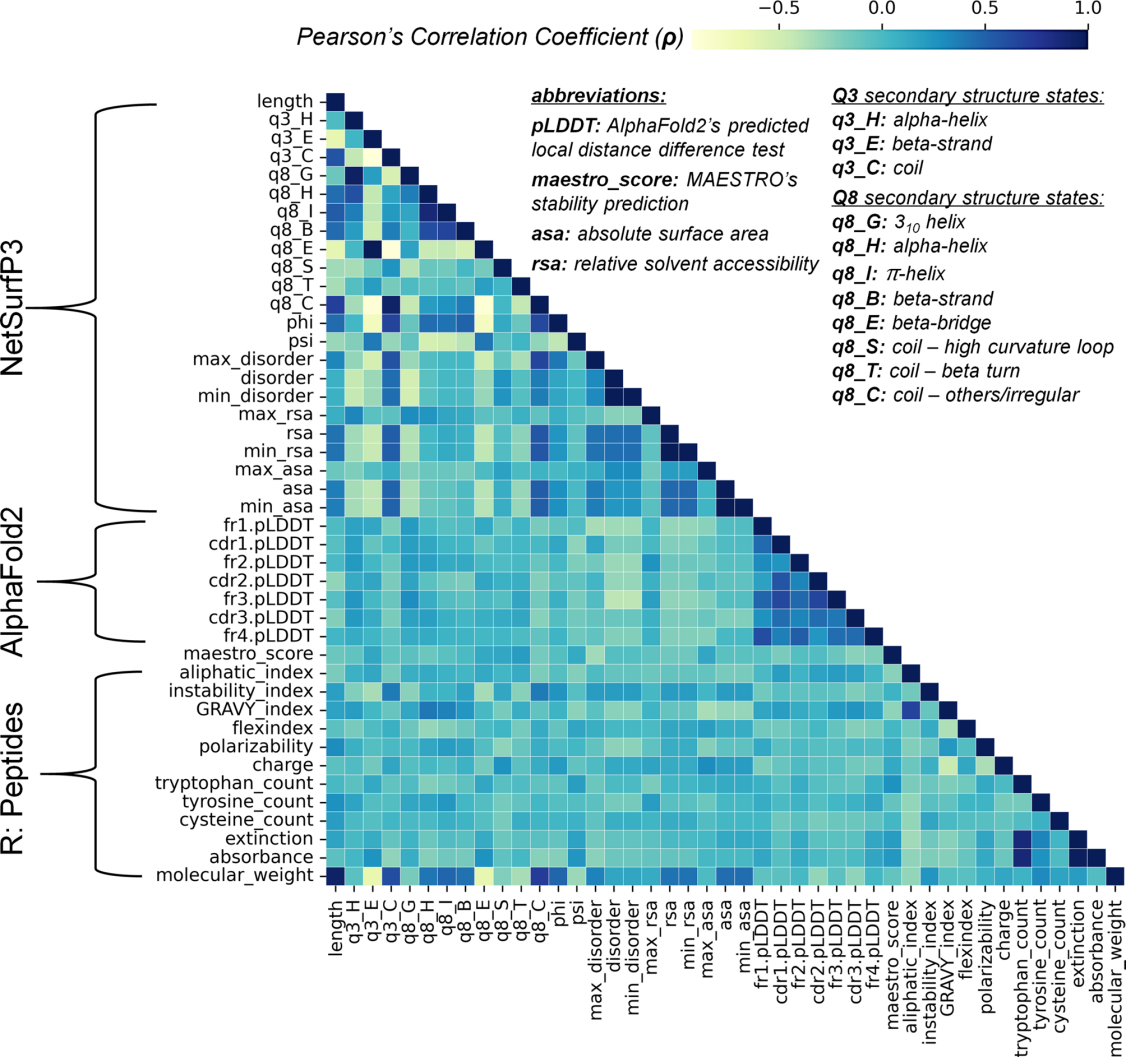
Correlations between biophysical properties of nanobodies. Predicted values from NetSurfP3 display varying degrees of correlation with coil states (q3_C and q8_C) exhibiting high positive relationships with other structural features. AlphaFold2 pLDDT scores per region only exhibit relationships with each other, but minimal with other properties. Physicochemical characteristics from the R Peptides package have relatively low presence amongst other predicted biophysical properties of nanobodies with the exception of molecular weight and number of tryptophan in a nanobody sequence.

Preliminary exploratory analyses were conducted using cross-correlations between features to identify relationships between the nanobodies’ biophysical properties (due to the large, unlabeled metadata of protein embeddings, these were excluded in cross-correlations in Fig. 2). NetSurfP3 predictions mostly show positive correlation, but it is interesting to note that q3_C and q8_C coil structure states are highly correlated with the phi (N-Cα) torsion angles, disorder, relative solvent accessibility (rsa), and absolute surface area (asa) values (approximately ρ > 0.5). It was reported by Kurgan et al.^72^ that the predicted secondary structure using 1-dimensional descriptors of protein structure is useful in the prediction of disorder, flexible region, fold recognition and relative solvent accessibility. It was also previously observed that the backbone torsion angle and secondary structures of a protein are highly correlated^73^. In this context, the predicted coil states contribute to higher correlation of these structural properties as compared with alpha helices and beta strands. The intricacies of FR/CDR regions of nanobodies are instrumental in their sequence identity and arrangement such as the conserved frameworks (FR1–4) and variable CDRs, especially in the context of their thermostability (please refer to^74^ for review), thus we see a slight cross-correlation with AlphaFold2’s pLDDTs scores. On another note, the predicted physicochemical characteristics (i.e., maestro_score, aliphatic index, and others) show low correlation with any of the features, with the exception of (1) molecular weight being highly correlated primarily with sequence length (ρ = 0.94), q3_C (ρ = 0.62), q8_C (ρ = 0.71), phi (ρ = 0.49), rsa (ρ = 0.42), and asa (ρ = 0.45); and (2) number of tryptophan in the sequence highly correlated with extinction (ρ = 0.87) and absorbance (ρ = 0.88) as compared with tyrosine and cysteine. In further investigating these cross-correlations, these features’ predictive capabilities are further discussed below.

It may seem counterintuitive that the physicochemical properties of nanobodies barely contribute a relationship to each other. For example, Natesan et al.^75^ reported that in the estimations of the solvent-accessible surface for individual cysteines in IgG1 antibody through molecular dynamics simulations, the interchange and hinge cysteines have > 1000 higher solvent accessibility as compared to intrachain cysteines. They suggest that the cysteine’s accessibility to the surrounding solvent is one of the primary determinants of its disulfide bond stability. By this notion, although unrelated to estimating an antibody’s melting temperature, the number of cysteines or any quantity that involves this amino acid should be at least correlated to any other biophysical property. However, Fig. 2 clearly shows that the number of cysteines is minimally correlated across all the features. Consequently, since the nanobody regions or antibody numbering are mainly governed by the positions of cysteine residues, we have also explored their pLDDT scores for each region and their relationship to their corresponding T_m_s.

### Framework and complementarity-determining regions show extremely low correlation to nanobody thermostability using AlphaFold2 pLDDT scores

**Figure 3:**
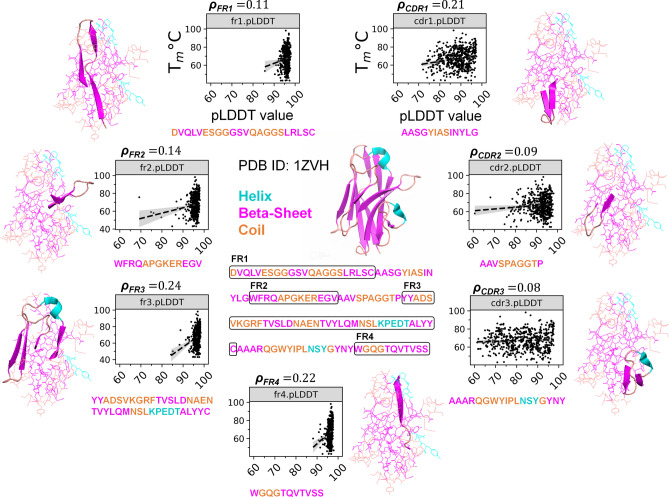
AlphaFold2 pLDDT scores per nanobody region vs T_m_s (°C). Framework regions’ pLDDT scores are generally more correlated with T_m_s. Linear fit regression lines (dashed line with gray shades) were added through Seaborn module’s *regplot* function. 1ZVH nanobody was rendered using PyMOL software.

In light of the AlphaFold2’s breakthrough in protein structure prediction, recent studies have used AlphaFold2’s pLDDT scores for testing discriminative capabilities for classification and even in the context of analyzing antibody-antigen interface residue scores^76,77^. All 567 nanobodies’ structural representations were predicted using AlphaFold2 for consistency where each processed nanobody has an output PDB file (included in the GitHub repository). Regarding the importance of cysteine residue positions in T_m_s of nanobodies, Saerens et al.^78^ investigated a VHH that naturally had an extra pair of cysteines at positions Cys54 and Cys78 which led to a disulfide bond linking FR2 and FR3. Moreover, several studies have also demonstrated that introducing an additional disulfide bond into the hydrophobic core of llama VHHs between FR2 and FR3 resulted into an increased thermal stability at neutral pH, but also better resistance to proteolytic degradation^79–81^. Here, regional pLDDT scores of the 567 nanobodies display varying relationships with T_m_s wherein FR3 exhibits the highest correlation (ρ = 0.24) which suggests the importance of this region in thermostability (Fig. 3). Although there exists a measure of correlation between T_m_ and AlphaFold2 pLDDT scores, the overall analyses exhibit extremely low correlations for all nanobody regions. The results suggest that there is not enough information between regional AlphaFold2’s pLDDT scores and the nanobody thermostability to be of significant value for T_m_ prediction.

### Protein embeddings exhibit predictive qualities for nanobody thermostability

**Figure 4:**
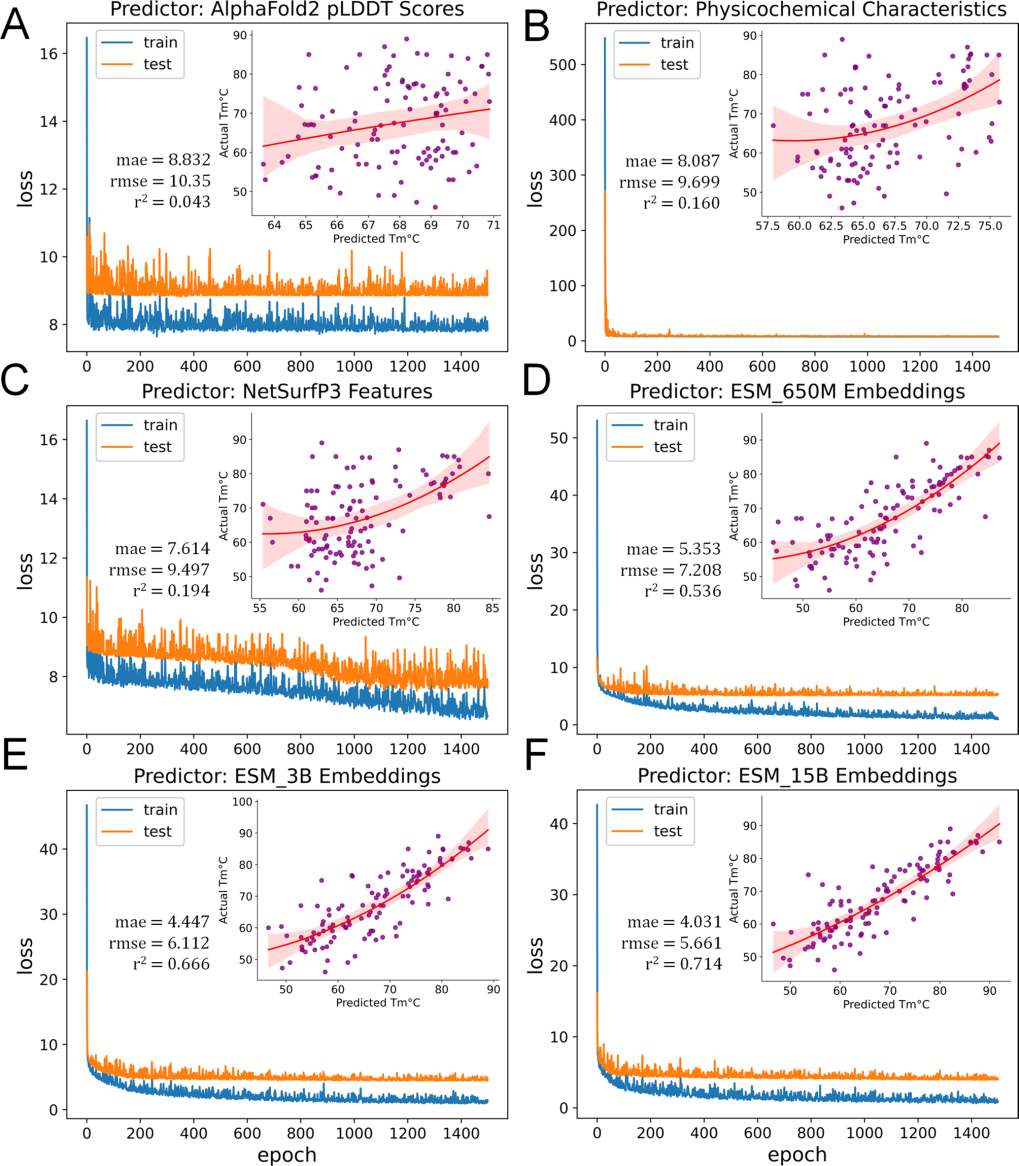
DNN model training. Predictive features using (**A**) AlphaFold2 pLDDT scores per region (FRs 1–4, CDRs 1–3), (**B**) nanobody physicochemical properties, and (**C**) structural predictions using NetSurfP3. (**D–F**) Protein embeddings using pre-trained ESM models. Predicted vs Actual T_m_°C denoted as scatterplots (purple dots) with second order polynomial regression lines (red) at 99% confidence intervals (red shades).

Protein language models in current literature are of growing interest in contexts of protein stability changes, protein sequencing/scaffold filling, and other biophysical property predictions^54,82,83^. Recently, protein representations or embeddings have been used for protein engineering, such as predicted solubility, thermophilicity, and other property prediction or classification tasks^19,23,24^. As the nanobodies’ biophysical properties have displayed varying degrees of correlation depicted in Figs. 2 and 3, we have used each of these computed predictive features and compared each predicted protein structure embedding from the ESM models, in estimating nanobody thermostability (Fig. 4).

Due to the large number of predictors, we have initially opted to use a deep neural network (DNN) model using Keras Sequential API with 1 input layer, 3 hidden layers, and 1 output layer, to accommodate dataset complexities. Additionally, we have compared different lengths of model training and determined that the best-performing training time equal to 1500 epochs (model training diagnostics can also be found in the GitHub repository). Thus, for consistency, we applied the same parameters to all predictive features in the dataset.

Preliminary results for DNN training reveal that protein embeddings exhibit better predictive capabilities than AlphaFold2 pLDDT scores (Fig. 4A), the nanobodies’ physicochemical characteristics (Fig. 4B), and structural predictions from NetSurfP3 (Fig. 4C). Each of the pre-trained ESM models (Fig. 4D–F), overall, have better predictions with the ESM_15B Embeddings as the best-performing predictive feature (Fig. 4F) with a mean absolute error (mae) of 4.03 °C, root mean-squared error (rmse) of 5.66 °C, and coefficient of determination (r^2^) of 71.4% validated on the test dataset. The second-order polynomial regression lines (in red) and purple scatterplots of T_m_s also indicate a better fit as compared with the other predictors.

### DNN performs better than other machine learning techniques using protein embeddings

**Figure 5:**
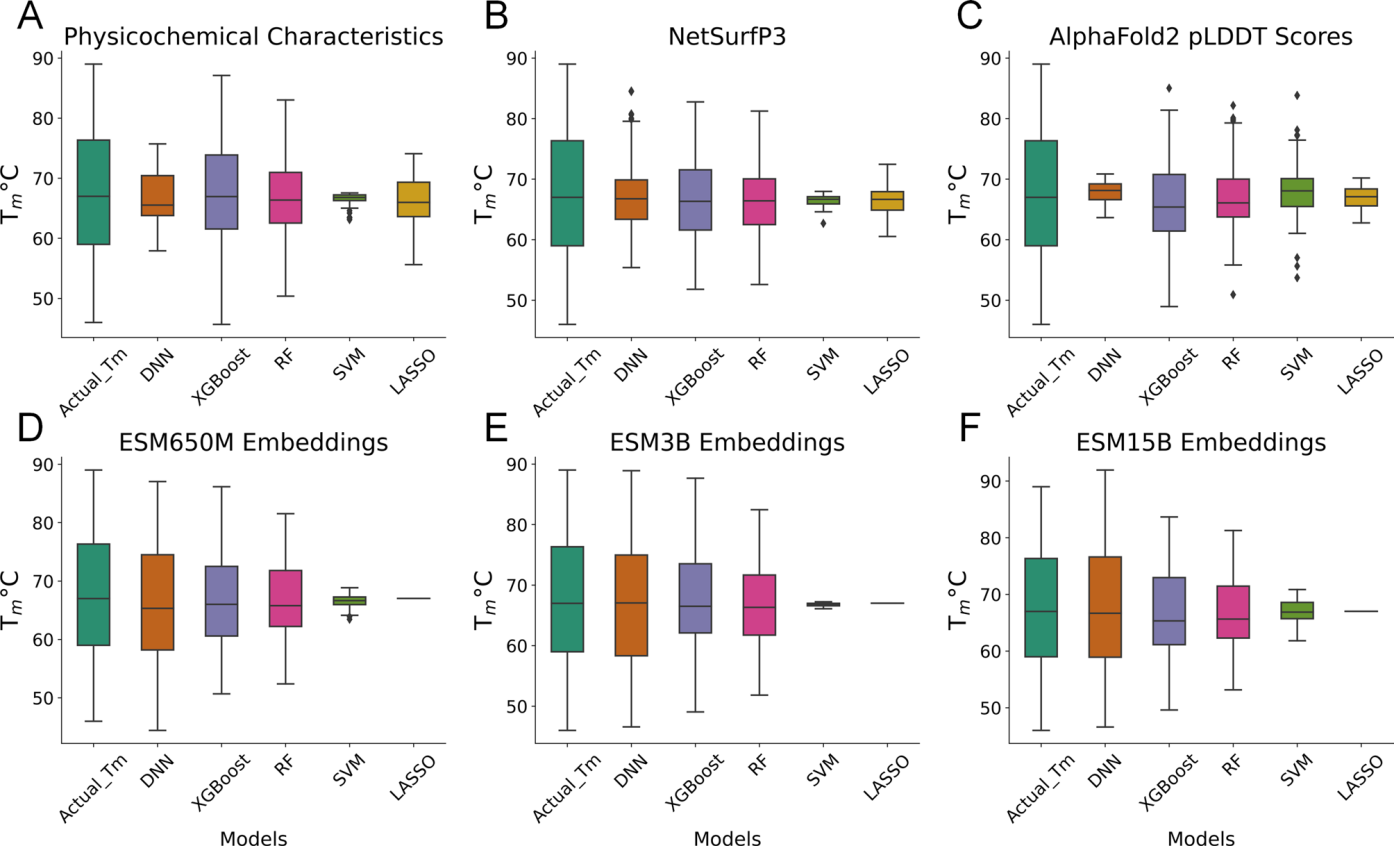
Nanobody feature performance by multiple machine learning models. Similar to previous analyses, various nanobody features were tested amongst several ML techniques to investigate predictive performance. (**A–C**) XGBoost and Random Forests perform better than DNN, SVM, and LASSO using a lower number of predictors; while (**D–F**) DNN performs better using ESM embeddings which contain thousands of columns of nanobody representations.

Upon investigating the preliminary results of using DNN on various nanobody features, we have compared these same features on other appropriate machine learning models with a proven track record of high performance and validity, such as gradient boosting, decision trees, and multiple ensemble algorithms. Similar to the previous analyses, the nanobodies’ ESM embeddings outperformed all other features in estimating T_m_s (Fig. 5).

**Figure 6:**
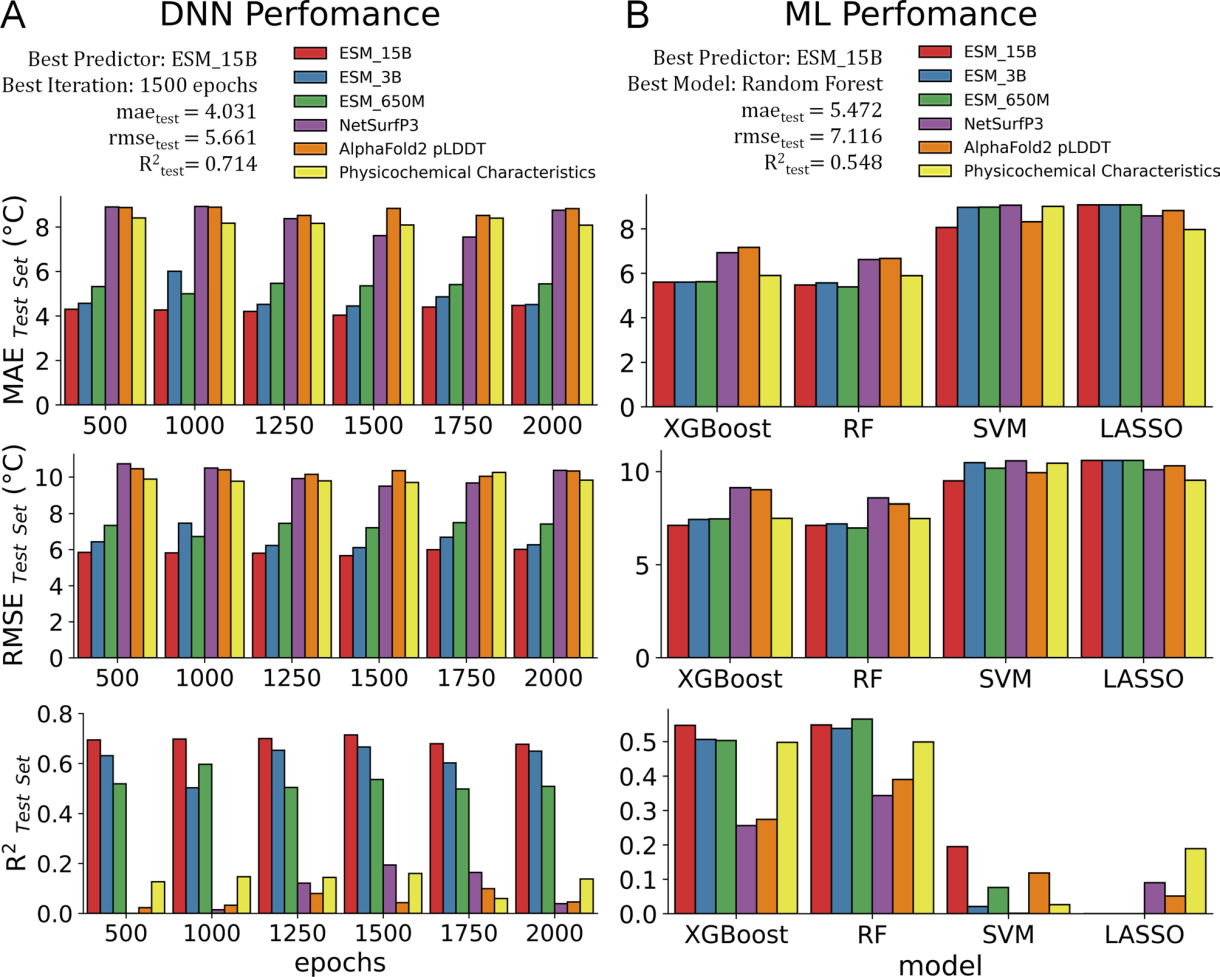
DNN vs ML performance. All performance metrics are validated using test data. (**A**) 1500 epochs displayed the best DNN performance with ESM_15B as the best predictor. (**B**) Random Forest outperforms all other ML models presented with a close tie with XGBoost.

However, it is important to note that using a lesser number of predictors (e.g., NetSurfP3 has 23 columns/features vs ESM embeddings ranging from 1280 to 5120 columns), XGBoost and Random Forest algorithms outperform DNN (Fig. 5A–C, 6b) while SVM and LASSO perform the worst. From this investigation, as the number of features grow larger and more complex, DNNs can provide a better T_m_ approximation than standard ML techniques (Fig. 6).

### Predictive performance of nanobody feature combinations

**Figure 7:**
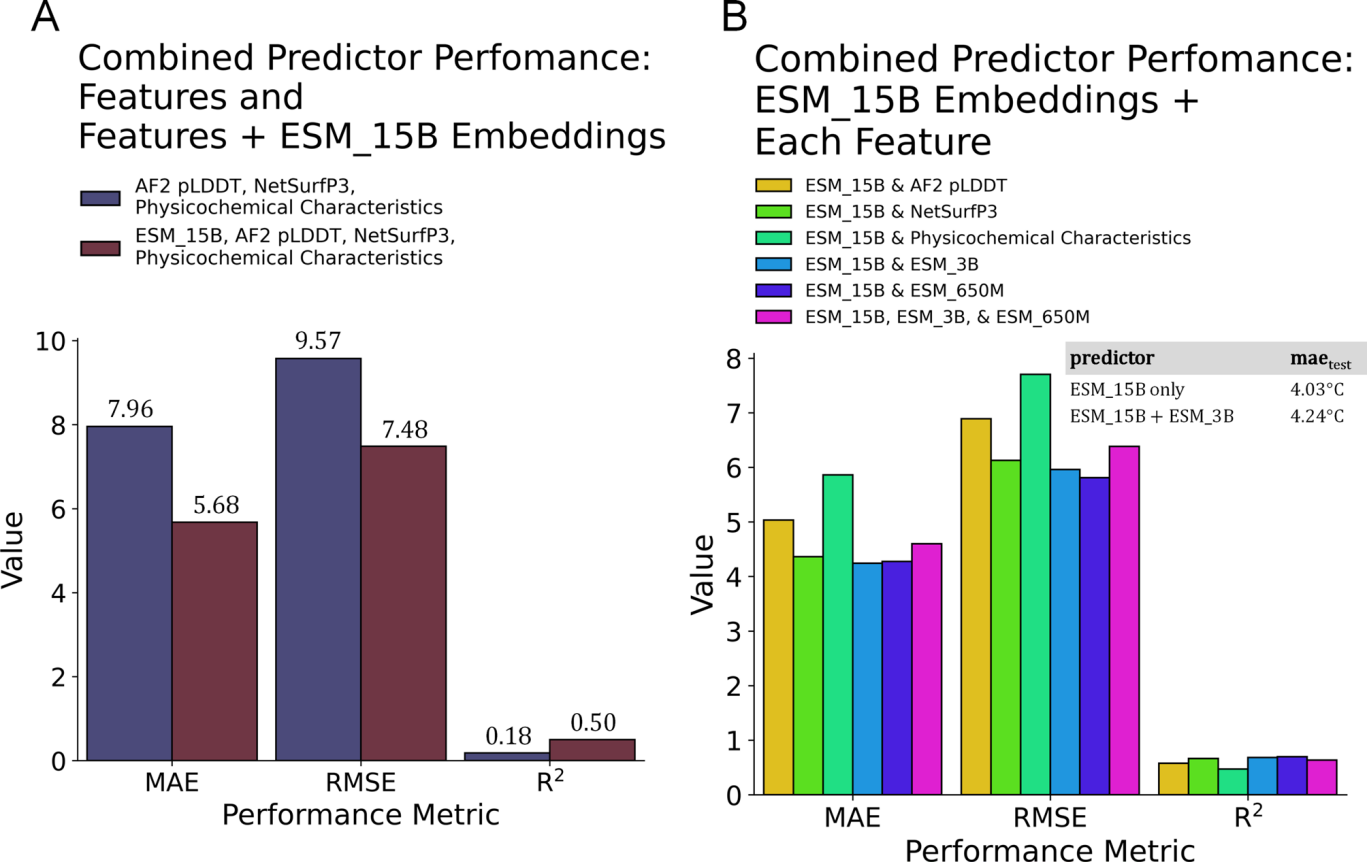
Predictive performance of nanobody feature combinations. (**A**) Combined sets of predictors increased in performance upon inclusion of ESM_15B embeddings. (**B**) Each combination the best-performing feature (ESM_15B) amongst other predictors. Solely using ESM_15B embeddings as the predictive feature outperforms all cases.

Using the best-performing model (i.e., DNN ESM_15B at 1500 epochs), we have used different permutations of predictors if combining the features can increase T_m_ estimations. We have combined AlphaFold pLDDT scores, NetSurfP3, and physicochemical characteristics of nanobodies along with ESM_15B embeddings. The mae improved by ~ 29%, rmse by ~ 22%, and coefficient of determination increased by 32%, all of which were validated using the test set (Fig. 7A). However, this is not the same for all cases—in permuting the ESM_15B embeddings with each of the selected features, no improvements were observed. In fact, combining the two best predictive features (i.e., ESM_15B and ESM_3B) resulted in higher mean absolute error (4.03 °C and 4.24 °C, respectively; Fig. 7B), albeit relatively close mae_test_ values.

### TEMPRO predicts nanobody thermostability accurately

**Figure 8:**
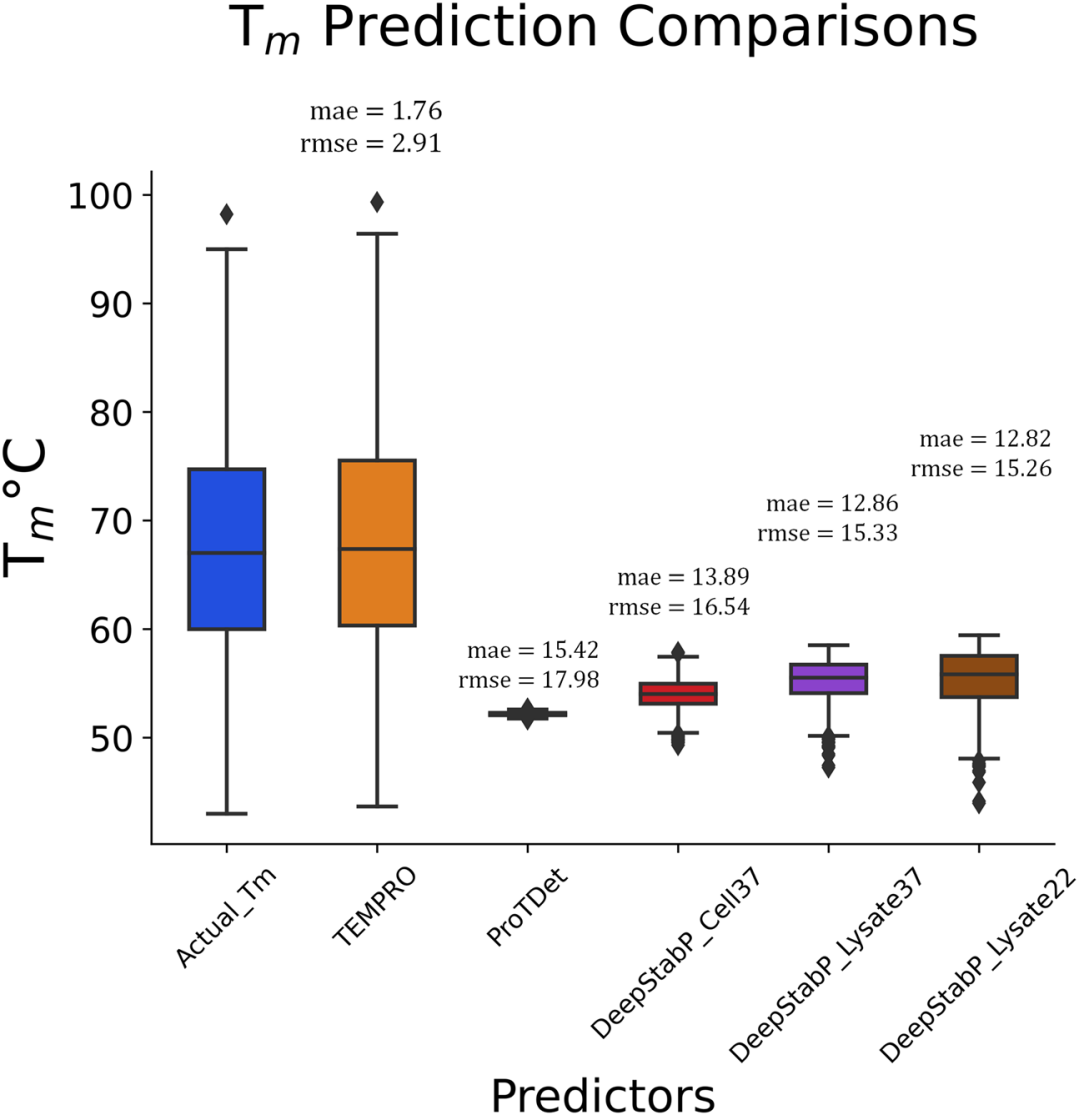
Melting temperature prediction comparisons with other online tools. TEMPRO (orange boxplot) outperforms other prediction tools (i.e., ProTDet and DeepStabP) and closely resemble the distribution of the actual nanobody T_m_s (blue).

To compare our model predictions, we have explored other known melting temperature predictors in literature that can process a relatively large amount of protein sequences (Ref.^19^ and https://github.com/GavrilenkoA/Tm_prediction) and not classifying the predictors into a set of ranges or thresholds (Refs.^22,23,84^ and https://github.com/zhibinlv/BertThermo). Since our model is specifically tailored to nanobodies, it outperforms the other T_m_ predictors (Fig. 8). For consistency, we used our saved DNN model to predict all the 567 nanobody sequences’ T_m_ instead of the test dataset. This has resulted in mae of 1.76 °C and rmse of 2.91 °C.

### Validation with another database

**Figure 9:**
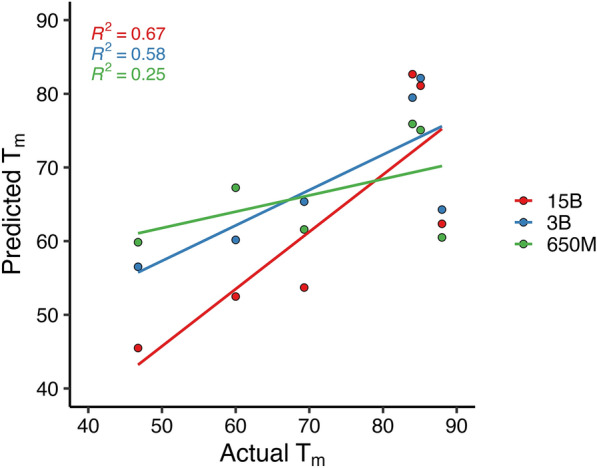
Experimental validation of nanobody T_m_s using pre-trained models from TEMPRO. The pre-trained ESM_15B parameter performs the best prediction in estimating the T_m_s of nanobody sequences not included in the NbThermo dataset with the highest coefficient of determination (R^2^ = 0.67). Actual T_m_ values are found in Table 4.

Furthermore, since NbThermo includes most of the nanobodies in current literature, the availability of data is scarce. To compare our pre-trained models with existing experimental data, several antibodies and their variants which contain experimentally-determined T_m_s were obtained from another database (https://research.naturalantibody.com/)^85^ that is separate from the existing dataset used for training and validation. The T_m_ prediction of the sequences using our three different pre-trained models are summarized in Table 4. For robustness, the sequences selected also displayed unusually low or high T_m_s ranging from ~ 46 to 88 °C (please see the reference column in Table 4). These experimental T_m_ values are also found in the GitHub repository (listed here upon acceptance). When comparing the ESM models’ results to the reference values using linear regression, ESM_15B is the best performer with an R^2^ of 0.67, followed by ESM_3B with 0.58, and ESM_650M with 0.25. Except for the predictions of the T_m_ of 4W68, where all models fared poorly, predictions made by ESM_15B and to a lesser extent 3B were relatively close to the reference T_m_s (Fig. 9). In contrast, the ProTDet and DeepStabP model predictions have no correlation with the reference T_m_s.

**Table 4:** Experimental validation of T_m_ prediction using pre-trained models (all units in °C).

PDB ID	Reference	Actual Tm	ESM_650M	ESM_3B	ESM_15B	ProTDet	DeepStabP lysate 22
4IDL	^86^	46.75 ± 1.6	59.85	56.52	45.49	52.39	54.38
4TYU	^87^	85.1 ± 0.1	75.08	82.13	81.10	52.34	57.04
4U05	^87^	84 ± 0.6	75.91	79.48	82.66	52.29	56.88
4W68	^87^	88 ± 4.0	60.50	64.28	62.35	52.19	56.75
4W70	^87^	60 ± 1.0	67.25	60.17	52.48	52.19	58.32
5SV3	^88^	69.3 ± 0.5	61.58	65.36	53.70	52.27	59.08

## Discussion

We have demonstrated the applicability of protein embeddings as a predictive feature of estimating nanobodies’ thermostability with a relatively high accuracy. This tool can help decrease the tedious and expensive production costs of manually deriving or synthesizing nanobodies to achieve or determine a desired property. VHHs can be remodeled, engineered and reassembled to generate new antibody-like molecules with desired properties and specificities, consisting of multiple units of the same or of different function^89^. As an example, Tomimoto et al. have previously demonstrated that a comprehensive mutagenesis approach exhibits low efficiency for identifying nanobody mutants with improved T_m_, but instead proposed a single mutation in VHH which can increase the T_m_ by more than 5 °C while nearly maintaining affinity^90^. Overall, appropriately engineered biomolecules should exhibit pharmacokinetic properties that make them desirable to both production and application, especially an initial estimate of their melting temperature.

### Comparisons of predictive features

Researchers have also previously demonstrated that the replacement of cysteine residues by appropriate amino acids will improve the heat resistance of a native VHH (please see^14^ for a comprehensive review). Multiple studies also showed the effects of having up to three disulfide bonds in one nanobody where the three bonds consist of the bond found in the wild type nanobody, a bond analogous and a novel bond connecting CDR1 and FR3^78,91^, resulting into stability improvements of up to 19 °C. It was also previously reported that moderate modifications to CDR1 that may improve the nanobody stability while preserving its binding capacity which suggests that CDR1 can be also a region of interest in thermostability improvements^92^. With this premise, we have used predictions from AlphaFold2 to explore its capabilities as a predictive feature for each of the nanobody regions. However, as the low correlations suggest from Fig. 3, other nanobody regional measures or metrics should be considered.

Although a considerable number of crystal structures of nanobodies have been registered in the Protein Data Bank, only few structures contain their T_m_s^18^. Additionally, new insights were gained by determining complexities of full-length antibodies from its early studies, such as from sharks, camelids, and humans to these single functional fragment antibodies. Basic biophysical properties such as amino acid lengths, hydrophobicity indices, cysteine counts, and instability indices of the nanobody sequences were preliminarily chosen as inspection tools for the bio-relevance of the predictors. The high stability of nanobodies indicated by the hydrophobicity, solvent accessibilities, and instability indices are well-sought biophysical properties which render their potential development as a therapeutic. Counter-intuitively, these features fail to increase the predictive power of machine learning techniques in estimating the nanobodies’ thermostability. Relatively, using protein embeddings offer such an alternative for better T_m_ estimation as demonstrated in this study.

### Protein embeddings, hardware limitations, and deep learning predictions

Protein embeddings are numerical representations of encoding protein properties and functions which are suited for multiple tasks^93–95^. Each model has its own strengths and weaknesses (such as speed, memory footprint, computational cost). It is generally recognized that there is no “one size fits all” model when it comes to a specific task, even with suggested hardware requirements. Aside from the other nanobody characteristics previously discussed, we generated the 650M (650 million parameters, 2.4GB), 3B (3 billion, 5.3 GB), and 15B (15 billion, 28.2GB) ESM embeddings for the 567 sequences using an NVIDIA H100 GPU (please refer to https://github.com/facebookresearch/esm for number of layers, embedding dimensions, and other information). Generating the 567 embeddings for the largest pre-trained model (ESM 15B) required approximately 5 h of runtime. In contrast, both training the models highlighted in this work and predicting a nanobody’s melting temperature complete in under a minute using an NVIDIA 2080 Ti GPU. These runtimes will vary depending on the hardware used.

Beyond their predictive value, embeddings can also enable the ability to highlight which amino acids are responsible for T_m_ changes, and through this capturing the nanobody’s T_m_ changes upon point mutation can realistically be achieved. Mapping the pre-trained model’s embeddings to specific residues could inform which residues, when altered, are most likely to yield a more thermostable nanobody. We therefore recommend this work for future research, as it could provide a mechanism for guiding small alterations that enhance nanobody thermostability, especially if paired with information that would allow for avoidance of point mutations that would negatively impact affinity and other desired attributes.

As modern biotechnology makes breakthroughs in protein sequence generation through generative deep learning methods, the prominent interest in antibody production has its merits. Multiple studies have demonstrated the feasibility of synthetically derived novel proteins through the use of deep-learning techniques^96–99^. Automated systems can process small or large sequence data through artificial neural networks that rely on many layers of nonlinear processing units for learning data representations. This architecture has been proven useful in protein synthesis molecular prediction algorithms—antimicrobial design, antibiotic discovery, and improved thermostability^83,97^. While such approaches continue to develop, these predictive techniques in determining biophysical properties of proteins from their sequence alone will continue to contribute to the design and evolution of synthetic biomolecules.

## Data Availability

All results in this study including pre-trained models, raw and processed datasets, and model training can be found in our GitHub repository (https://github.com/Jerome-Alvarez/TEMPRO). For further requests or inquiries, please feel free to contact the authors: Jerome Anthony E. Alvarez (Jerome.A.Alvarez.civ@us.navy.mil) or Scott N. Dean (Scott.N.Dean.civ@us.navy.mil).

## References

[1] Henry, K. A. & MacKenzie, C. R. Antigen recognition by single-domain antibodies: Structural latitudes and constraints. *MAbs* **10**(6), 815–826 (2018). 29916758 10.1080/19420862.2018.1489633 PMC6260137

[2] Wesolowski, J. *et al.* Single domain antibodies: Promising experimental and therapeutic tools in infection and immunity. *Med. Microbiol. Immunol.* **198**, 157–174 (2009). 19529959 10.1007/s00430-009-0116-7 PMC2714450

[3] Ventola, C. L. The antibiotic resistance crisis. *Pharm. Therap.* **40**(4), 277–283 (2015). PMC4378521 25859123

[4] Gould, I. M. & Bal, A. M. New antibiotic agents in the pipeline and how they can help overcome microbial resistance. *Virulence* **4**(2), 185–191 (2013). 23302792 10.4161/viru.22507 PMC3654619

[5] McConnell, A. D. *et al.* A general approach to antibody thermostabilization. *MAbs* **6**(5), 1274–1282 (2014). 25517312 10.4161/mabs.29680 PMC4623350

[6] Ward, E. S. *et al.* Binding activities of a repertoire of single immunoglobulin variable domains secreted from *Escherichia coli*. *Nature* **341**(6242), 544–546 (1989). 2677748 10.1038/341544a0

[7] Hamers-Casterman, C. *et al.* Naturally occurring antibodies devoid of light chains. *Nature* **363**(6428), 446–448 (1993). 8502296 10.1038/363446a0

[8] Ovchinnikov, V. *et al.* Role of framework mutations and antibody flexibility in the evolution of broadly neutralizing antibodies. *Elife* **7**, 1 (2018). 10.7554/eLife.33038 PMC5828663 29442996

[9] Kiguchi, Y. *et al.* The VH framework region 1 as a target of efficient mutagenesis for generating a variety of affinity-matured scFv mutants. *Sci. Rep.* **11**(1), 8201 (2021). 33859250 10.1038/s41598-021-87501-7 PMC8050046

[10] Nguyen, V. K. *et al.* Camel heavy-chain antibodies: Diverse germline V(H)H and specific mechanisms enlarge the antigen-binding repertoire. *EMBO J.* **19**(5), 921–930 (2000). 10698934 10.1093/emboj/19.5.921 PMC305632

[11] Muyldermans, S. *et al.* Sequence and structure of VH domain from naturally occurring camel heavy chain immunoglobulins lacking light chains. *Protein Eng.* **7**(9), 1129–1135 (1994). 7831284 10.1093/protein/7.9.1129

[12] Ding, L. *et al.* Structural insights into the mechanism of single domain VHH antibody binding to cortisol. *FEBS Lett.* **593**(11), 1248–1256 (2019). 31049949 10.1002/1873-3468.13398

[13] Rudolph, M. J. *et al.* Contribution of an unusual CDR2 element of a single domain antibody in ricin toxin binding affinity and neutralizing activity. *Protein Eng. Des. Select.* **31**(7–8), 277–287 (2018). 10.1093/protein/gzy022 PMC6277176 30265352

[14] Bever, C. S. *et al.* VHH antibodies: Emerging reagents for the analysis of environmental chemicals. *Anal. Bioanal. Chem.* **408**(22), 5985–6002 (2016). 27209591 10.1007/s00216-016-9585-x PMC4983233

[15] Polonelli, L. *et al.* Antibody complementarity-determining regions (CDRs) can display differential antimicrobial, antiviral and antitumor activities. *PLoS ONE* **3**(6), e2371 (2008). 18545659 10.1371/journal.pone.0002371 PMC2396520

[16] Liu, J. L. *et al.* Thermal stability and refolding capability of shark derived single domain antibodies. *Mol. Immunol.* **59**(2), 194–199 (2014). 24667069 10.1016/j.molimm.2014.02.014

[17] Kunz, P. *et al.* The structural basis of nanobody unfolding reversibility and thermoresistance. *Sci. Rep.* **8**(1), 7934 (2018). 29784954 10.1038/s41598-018-26338-z PMC5962586

[18] Bekker, G. J., Ma, B. & Kamiya, N. Thermal stability of single-domain antibodies estimated by molecular dynamics simulations. *Protein Sci.* **28**(2), 429–438 (2019). 30394618 10.1002/pro.3546 PMC6319760

[19] Jung, F. *et al.* DeepSTABp: A deep learning approach for the prediction of thermal protein stability. *Int. J. Mol. Sci.* **24**(8), 7444 (2023). 37108605 10.3390/ijms24087444 PMC10138888

[20] Li, M. *et al.* DeepTM: A deep learning algorithm for prediction of melting temperature of thermophilic proteins directly from sequences. *Comput. Struct. Biotechnol. J.* **21**, 5544–5560 (2023). 38034401 10.1016/j.csbj.2023.11.006 PMC10681957

[21] Yang, Y. *et al.* ProTstab2 for prediction of protein thermal stabilities. *Int. J. Mol. Sci.* **23**, 18 (2022). 10.3390/ijms231810798 PMC9505338 36142711

[22] Ku, T. *et al.* Predicting melting temperature directly from protein sequences. *Comput. Biol. Chem.* **33**(6), 445–450 (2009). 19896904 10.1016/j.compbiolchem.2009.10.002

[23] Haselbeck, F. *et al.* Superior protein thermophilicity prediction with protein language model embeddings. *NAR Genom. Bioinform.* **5**(4), 087 (2023). 10.1093/nargab/lqad087 PMC10566323 37829176

[24] Outeiral, C. & Deane, C. M. Codon language embeddings provide strong signals for use in protein engineering. *Nat. Mach. Intell.* **6**(2), 170–179 (2024). 10.1038/s42256-024-00791-0

[25] Valdés-Tresanco, M. S. *et al.* NbThermo: A new thermostability database for nanobodies. *Database* **2023**, 21 (2023). 10.1093/database/baad021 PMC10091358 37042467

[26] Kunz, P. *et al.* Exploiting sequence and stability information for directing nanobody stability engineering. *Biochim. Biophys. Acta Gen. Subj.* **1861**(9), 2196–2205 (2017). 28642127 10.1016/j.bbagen.2017.06.014 PMC5548252

[27] Osorio, D., Rondón-Villarreal, P. & Torres, R. Peptides: A package for data mining of antimicrobial peptides. *R J.* **7**(1), 4–14 (2015). 10.32614/RJ-2015-001

[28] Ikai, A. Thermostability and aliphatic index of globular proteins. *J. Biochem.* **88**(6), 1895–1898 (1980). 7462208

[29] Guruprasad, K., Reddy, B. V. & Pandit, M. W. Correlation between stability of a protein and its dipeptide composition: A novel approach for predicting in vivo stability of a protein from its primary sequence. *Protein Eng.* **4**(2), 155–161 (1990). 2075190 10.1093/protein/4.2.155

[30] Kyte, J. & Doolittle, R. F. A simple method for displaying the hydropathic character of a protein. *J. Mol. Biol.* **157**(1), 105–132 (1982). 7108955 10.1016/0022-2836(82)90515-0

[31] Bannas, P., Hambach, J. & Koch-Nolte, F. Nanobodies and nanobody-based human heavy chain antibodies as antitumor therapeutics. *Front. Immunol.* **8**, 1 (2017). 29213270 10.3389/fimmu.2017.01603 PMC5702627

[32] Bhaskaran, R. & Ponnuswamy, P. K. Positional flexibilities of amino acid residues in globular proteins. *Int. J. Peptide Protein Res.* **32**(4), 241–255 (1988). 10.1111/j.1399-3011.1988.tb01258.x 6480218

[33] Dong, Y.-W. *et al.* Structural flexibility and protein adaptation to temperature: Molecular dynamics analysis of malate dehydrogenases of marine molluscs. *Proc. Natl. Acad. Sci.* **115**(6), 1274–1279 (2018). 29358381 10.1073/pnas.1718910115 PMC5819447

[34] Sheriff, S. *et al.* Influence of solvent accessibility and intermolecular contacts on atomic mobilities in hemerythrins. *Proc. Natl. Acad. Sci.* **82**(4), 1104–1107 (1985). 3856249 10.1073/pnas.82.4.1104 PMC397202

[35] Sandberg, M. *et al.* New chemical descriptors relevant for the design of biologically active peptides. A multivariate characterization of 87 amino acids. *J. Med. Chem.* **41**(14), 2481–2491 (1998). 9651153 10.1021/jm9700575

[36] Tesfaye, D. Y. *et al.* Targeting conventional dendritic cells to fine-tune antibody responses. *Front. Immunol.* **10**, 1529 (2019). 31333661 10.3389/fimmu.2019.01529 PMC6620736

[37] Pervez, S. *et al.* Effect of polarity and differentiation on antibody localization in multicellular tumour spheroid and xenograft models and its potential importance for in vivo immunotargeting. *Int. J. Cancer* **44**(5), 940–947 (1989). 2583872 10.1002/ijc.2910440532

[38] Wang, Y. *et al.* Investigation of the small size of nanobodies for a sensitive fluorescence polarization immunoassay for small molecules: 3-Phenoxybenzoic acid, an exposure biomarker of pyrethroid insecticides as a model. *J. Agric. Food Chem.* **67**(41), 11536–11541 (2019). 31589045 10.1021/acs.jafc.9b04621 PMC7134064

[39] Moore, D. S. Amino acid and peptide net charges: A simple calculational procedure. *Biochem. Educ.* **13**(1), 10–11 (1985). 10.1016/0307-4412(85)90114-1

[40] Lehninger, A. L. *Lehninger Principles of Biochemistry* 6th edn. (W.H. Freeman, 2013).

[41] Rabia, L. A. *et al.* Net charge of antibody complementarity-determining regions is a key predictor of specificity. *Protein Eng. Des. Select.* **31**(11), 409 (2018). 10.1093/protein/gzz002 PMC6524611 30770934

[42] Frank, S. A. *Specificity and Cross-Reactivity, in Immunology and Evolution of Infectious Disease* (Princeton University Press, 2002). 20821852

[43] Ghisaidoobe, A. B. & Chung, S. J. Intrinsic tryptophan fluorescence in the detection and analysis of proteins: A focus on Förster resonance energy transfer techniques. *Int. J. Mol. Sci.* **15**(12), 22518–22538 (2014). 25490136 10.3390/ijms151222518 PMC4284722

[44] Goldman, E. R. *et al.* Enhancing stability of camelid and shark single domain antibodies: An overview. *Front. Immunol.* **8**, 1 (2017). 28791022 10.3389/fimmu.2017.00865 PMC5524736

[45] Meitzler, J. L. *et al.* Conserved cysteine residues provide a protein-protein interaction surface in dual oxidase (DUOX) proteins. *J. Biol. Chem.* **288**(10), 7147–7157 (2013). 23362256 10.1074/jbc.M112.414797 PMC3591624

[46] Wilkins, M. R. *et al.* Protein identification and analysis tools in the ExPASy server. *Methods Mol. Biol.* **112**, 531–552 (1999). 10027275 10.1385/1-59259-584-7:531

[47] Simonian, M. H. Spectrophotometric determination of protein concentration. *Curr. Protoc. Toxicol.* **1**, 1–7 (2004). 10.1002/0471140856.txa03gs21 20976674

[48] Maity, H. *et al.* Comparison of predicted extinction coefficients of monoclonal antibodies with experimental values as measured by the Edelhoch method. *Int. J. Biol. Macromol.* **77**, 260–265 (2015). 25819219 10.1016/j.ijbiomac.2015.03.027

[49] Holt, L. J. *et al.* Domain antibodies: Proteins for therapy. *Trends Biotechnol.* **21**(11), 484–490 (2003). 14573361 10.1016/j.tibtech.2003.08.007

[50] Laimer, J. *et al.* MAESTRO—Multi agent stability prediction upon point mutations. *BMC Bioinform.* **16**(1), 116 (2015). 10.1186/s12859-015-0548-6 PMC4403899 25885774

[51] Høie, M. H. *et al.* NetSurfP-3.0: Accurate and fast prediction of protein structural features by protein language models and deep learning. *Nucleic Acids Res.* **50**(W1), W510–W515 (2022). 35648435 10.1093/nar/gkac439 PMC9252760

[52] Cohen, T., Halfon, M. & Schneidman-Duhovny, D. NanoNet: Rapid and accurate end-to-end nanobody modeling by deep learning. *Front. Immunol.* **13**, 958584 (2022). 36032123 10.3389/fimmu.2022.958584 PMC9411858

[53] Ruffolo, J. A. & Gray, J. J. Fast, accurate antibody structure prediction from deep learning on massive set of natural antibodies. *Biophys. J.* **121**(3), 155–156 (2022). 10.1016/j.bpj.2021.11.1942 PMC10129313 37185622

[54] Lin, Z. *et al.* Evolutionary-scale prediction of atomic-level protein structure with a language model. *Science* **379**(6637), 1123–1130 (2023). 36927031 10.1126/science.ade2574

[55] Wu, R. *et al.* High-resolution de novo structure prediction from primary sequence. *BioRxiv* **21**, 500999 (2022).

[56] AlQuraishi, M. Machine learning in protein structure prediction. *Curr. Opin. Chem. Biol.* **65**, 1–8 (2021). 34015749 10.1016/j.cbpa.2021.04.005

[57] Jumper, J. *et al.* Highly accurate protein structure prediction with AlphaFold. *Nature* **596**(7873), 583–589 (2021). 34265844 10.1038/s41586-021-03819-2 PMC8371605

[58] Valdés-Tresanco, M. S. *et al.* Structural modeling of nanobodies: A benchmark of state-of-the-art artificial intelligence programs. *Molecules* **28**(10), 3991 (2023). 37241731 10.3390/molecules28103991 PMC10220908

[59] Honegger, A. & Plückthun, A. Yet another numbering scheme for immunoglobulin variable domains: An automatic modeling and analysis tool. *J. Mol. Biol.* **309**(3), 657–670 (2001). 11397087 10.1006/jmbi.2001.4662

[60] Dunbar, J. & Deane, C. M. ANARCI: Antigen receptor numbering and receptor classification. *Bioinformatics* **32**(2), 298–300 (2015). 26424857 10.1093/bioinformatics/btv552 PMC4708101

[61] Apweiler, R. *et al.* UniProt: The universal protein knowledgebase. *Nucleic Acids Res.* **32**, 115–119 (2004). 14681372 10.1093/nar/gkh131 PMC308865

[62] Pedregosa, F. *et al.* Scikit-learn: Machine learning in python. *J. Mach. Learn. Res.* **12**, 2825–2830 (2011).

[63] Chen, T. & Guestrin, C. XGBoost: A scalable tree boosting system. In *Proc. 22nd ACM SIGKDD International Conference on Knowledge Discovery and Data Mining* 785–794 (Association for Computing Machinery, 2016).

[64] Breiman, L. Random Forests. *Mach. Learn.* **45**(1), 5–32 (2001). 10.1023/A:1010933404324

[65] Hearst, M. A. *et al.* Support vector machines. *IEEE Intell. Syst. Appl.* **13**(4), 18–28 (1998). 10.1109/5254.708428

[66] Tibshirani, R. Regression shrinkage and selection via the lasso. *J. R. Stat. Soc. Ser. B (Methodol.)* **58**(1), 267–288 (1996). 10.1111/j.2517-6161.1996.tb02080.x

[67] Rumelhart, D. E., Hinton, G. E. & Williams, R. J. Learning representations by back-propagating errors. *Nature* **323**(6088), 533–536 (1986). 10.1038/323533a0

[68] LeCun, Y., Bengio, Y. & Hinton, G. Deep learning. *Nature* **521**(7553), 436–444 (2015). 26017442 10.1038/nature14539

[69] Hinton, G. E. & Salakhutdinov, R. R. Reducing the dimensionality of data with neural networks. *Science* **313**(5786), 504–507 (2006). 16873662 10.1126/science.1127647

[70] Chollet, F. *Deep Learning with Python* (Simon and Schuster, 2021).

[71] Waskom, M. Seaborn: Statistical data visualization. *J. Open Source Softw.* **6**, 3021 (2021). 10.21105/joss.03021

[72] Kurgan, L. & Miri Disfani, F. Structural protein descriptors in 1-dimension and their sequence-based predictions. *Curr. Protein Peptide Sci.* **12**(6), 470–489 (2011). 21787299 10.2174/138920311796957711

[73] Singh, H., Singh, S. & Raghava, G. P. Evaluation of protein dihedral angle prediction methods. *PLoS ONE* **9**(8), e105667 (2014). 25166857 10.1371/journal.pone.0105667 PMC4148315

[74] Jin, B. K. *et al.* NANOBODIES®: A review of diagnostic and therapeutic applications. *Int. J. Mol. Sci.* **24**, 6 (2023). 10.3390/ijms24065994 PMC10057852 36983063

[75] Natesan, R. *et al.* Heterogeneity in disulfide bond reduction in IgG1 antibodies is governed by solvent accessibility of the cysteines. *Antibodies* **12**(4), 83 (2023). 38131805 10.3390/antib12040083 PMC10741012

[76] Yin, R. *et al.* Benchmarking AlphaFold for protein complex modeling reveals accuracy determinants. *Protein Sci.* **31**(8), e4379 (2022). 35900023 10.1002/pro.4379 PMC9278006

[77] Yin, R. & Pierce, B. G. Evaluation of AlphaFold antibody–antigen modeling with implications for improving predictive accuracy. *Protein Sci.* **33**(1), e4865 (2024). 38073135 10.1002/pro.4865 PMC10751731

[78] Saerens, D. *et al.* Disulfide bond introduction for general stabilization of immunoglobulin heavy-chain variable domains. *J. Mol. Biol.* **377**(2), 478–488 (2008). 18262543 10.1016/j.jmb.2008.01.022

[79] Zabetakis, D. *et al.* Evaluation of disulfide bond position to enhance the thermal stability of a highly stable single domain antibody. *PLoS ONE* **9**(12), e115405 (2014). 25526640 10.1371/journal.pone.0115405 PMC4272287

[80] Hussack, G. *et al.* Engineered single-domain antibodies with high protease resistance and thermal stability. *PLoS ONE* **6**(11), e28218 (2011). 22140551 10.1371/journal.pone.0028218 PMC3227653

[81] Tabares-da Rosa, S. *et al.* Competitive selection from single domain antibody libraries allows isolation of high-affinity antihapten antibodies that are not favored in the llama immune response. *Anal. Chem.* **83**(18), 7213–7220 (2011). 21827167 10.1021/ac201824z PMC3193053

[82] Sturtz, J. *et al*. Deep learning approaches for the protein scaffold filling problem. In *2022 IEEE 34th International Conference on Tools with Artificial Intelligence (ICTAI)* (2022).

[83] Stokes, J. M. *et al.* A deep learning approach to antibiotic discovery. *Cell* **180**(4), 688–702 (2020). 32084340 10.1016/j.cell.2020.01.021 PMC8349178

[84] Pudžiuvelytė, I. *et al.* TemStaPro: Protein thermostability prediction using sequence representations from protein language models. *Bioinformatics* **40**, 4 (2024). 10.1093/bioinformatics/btae157 PMC11001493 38507682

[85] Deszyński, P. *et al.* INDI—Integrated nanobody database for immunoinformatics. *Nucleic Acids Res.* **50**(D1), D1273–D1281 (2021). 10.1093/nar/gkab1021 PMC8728276 34747487

[86] Legler, P. M. *et al.* Structure of a low-melting-temperature anti-cholera toxin: llama V(H)H domain. *Acta Crystallogr Sect. F Struct. Biol. Cryst. Commun.* **69**, 90–93 (2013). 23385744 10.1107/S1744309112050750 PMC3564605

[87] George, J. *et al.* Structural and mutational analysis of a monomeric and dimeric form of a single domain antibody with implications for protein misfolding. *Proteins Struct. Funct. Bioinform.* **82**(11), 3101–3116 (2014). 10.1002/prot.24671 25136772

[88] Legler, P. M. *et al.* Stability of isolated antibody-antigen complexes as a predictive tool for selecting toxin neutralizing antibodies. *mAbs* **9**(1), 43–57 (2017). 27660893 10.1080/19420862.2016.1236882 PMC5240650

[89] Krah, S. *et al.* Single-domain antibodies for biomedical applications. *Immunopharmacol. Immunotoxicol.* **38**(1), 21–28 (2016). 26551147 10.3109/08923973.2015.1102934

[90] Tomimoto, Y., Yamazaki, R. & Shirai, H. Increasing the melting temperature of VHH with the in silico free energy score. *Sci. Rep.* **13**(1), 4922 (2023). 36966210 10.1038/s41598-023-32022-8 PMC10039853

[91] Hagihara, Y., Mine, S. & Uegaki, K. Stabilization of an immunoglobulin fold domain by an engineered disulfide bond at the buried hydrophobic region. *J. Biol. Chem.* **282**(50), 36489–36495 (2007). 17932041 10.1074/jbc.M707078200

[92] Orlando, M. *et al.* CDR1 Composition can affect nanobody recombinant expression yields. *Biomolecules* **11**, 9 (2021). 10.3390/biom11091362 PMC8465892 34572576

[93] Yang, K. K. *et al.* Learned protein embeddings for machine learning. *Bioinformatics* **34**(15), 2642–2648 (2018). 29584811 10.1093/bioinformatics/bty178 PMC6061698

[94] Yeung, W. *et al.* Tree visualizations of protein sequence embedding space enable improved functional clustering of diverse protein superfamilies. *Brief. Bioinform.* **24**, 1 (2023). 10.1093/bib/bbac619 PMC9851311 36642409

[95] Littmann, M. *et al.* Protein embeddings and deep learning predict binding residues for various ligand classes. *Sci. Rep.* **11**(1), 23916 (2021). 34903827 10.1038/s41598-021-03431-4 PMC8668950

[96] Ferruz, N., Schmidt, S. & Höcker, B. ProtGPT2 is a deep unsupervised language model for protein design. *Nat. Commun.* **13**(1), 4348 (2022). 35896542 10.1038/s41467-022-32007-7 PMC9329459

[97] Dean, S. N. *et al.* PepVAE: Variational autoencoder framework for antimicrobial peptide generation and activity prediction. *Front. Microbiol.* **12**, 725727 (2021). 34659152 10.3389/fmicb.2021.725727 PMC8515052

[98] Saka, K. *et al.* Antibody design using LSTM based deep generative model from phage display library for affinity maturation. *Sci. Rep.* **11**(1), 5852 (2021). 33712669 10.1038/s41598-021-85274-7 PMC7955064

[99] Humpe, A. & Peipp, M. Antibody engineering—Tailor-made next generation antibodies by molecular design. *Transfus Med. Hemother.* **44**(5), 290–291 (2017). 29070973 10.1159/000479617 PMC5649259

